# Identifying Potential Determinants of Faecal Contamination on Domestic Floors in Three Settings in Rural Kenya: A Mixed Methods Analysis

**DOI:** 10.1177/11786302241246454

**Published:** 2024-05-10

**Authors:** Hugo Legge, Karisa Kazungo, Sharon Muli, Lynne Elson, Jacinta Mwongeli, Katherine E Halliday, Victoria Ochwal, William Oswald, Robert Dreibelbis, Doris Njomo, Charles Mwandawiro, Ulrike Fillinger, Rachel Pullan, Stella Kepha

**Affiliations:** 1 London School of Hygiene & Tropical Medicine, UK; 2 International Centre of Insect Physiology and Ecology, Nairobi, Kenya; 3 KEMRI-Wellcome Trust, Nairobi, Kenya; 4 Centre for Tropical Medicine and Global Health, Nuffield Department of Medicine, University of Oxford, UK; 5 Jomo Kenyatta University of Agriculture and Technology, Nairobi, Kenya; 6 Global Health Division, International Development Group, RTI International, Research Triangle Park, NC, USA; 7 Eastern and Southern Africa Centre of International Parasite Control, Kenya Medical Research Institute, Nairobi, Kenya

**Keywords:** Enteric infections, flooring, soil-transmitted helminths, faecal contamination, Kenya, mixed methods, environmental health, housing, housing and health

## Abstract

Observational evidence suggests that household floors may be an important domain for the transmission of enteric and parasitic infections. However, little work has been done to investigate how household floors can become contaminated with human and animal faeces. This study uses a mixed methods approach to postulate the proximal and distal determinants of household floor contamination with faeces in groups of rural villages in 3 counties in Kenya (Bungoma, Kwale and Narok). Quantitative data was collected through a household census and analysed descriptively and using mixed effects logistic regression models. Qualitative data was collected through unstructured observations of daily routines and in-depth interviews. These data were analysed thematically with case memos produced for routine activities that were hypothesised to be determinants of floor contamination. Possible proximal determinants of floor contamination included; (1) animal contact with floors; (2) child faeces disposal, and; (3) floor cleaning routines. Distal determinants are suggested to be rooted in the socioeconomic, environmental, and cultural context in which households were located and included; (1) the type and number of animals owned by households; (2) presence/absence of dedicated shelters for housing animals at night, which impacted whether sleeping or cooking areas were exposed to animals; (3) Accessibility of inside spaces to poultry and other roaming animals; (4) ownership of an improved floor; (5) ability of animals to access neighbours compounds; (6) seasonal changes in weather. These results will be of use in identifying the contexts in which faecal contamination of domestic floors may be contributing towards transmission of enteric and parasitic infections and in designing effective interventions to prevent this exposure.

## Background

Enteric and parasitic infections are among the leading causes of global child morbidity and mortality.^1-3^ Diarrhoea resulting from such infections is responsible for 446 000 deaths annually,^3^ while associated anaemia and malnutrition can result in stunting (impaired physical growth), diminished cognitive development, arrested educational attainment, and reduced psychological wellbeing and quality of life.^4-8^

Water, sanitation and hygiene (WASH) interventions are one of the primary approaches used by disease control programmes to prevent diarrhoeal diseases and some parasitic infections such as soil-transmitted helminthiasis (STH). However, mixed results from recent large-scale, high-fidelity WASH trials suggest that transmission of diarrhoea-causing pathogens can persist along pathways unaffected by standard, traditional WASH interventions – improved water quality or quantity, improved sanitation, and improved handwashing with soap at key moments.^9-11^

Household floors present a convenient surface on which many pathogens can survive and proliferate before being directly or indirectly brought into contact with a new host. Environmental sampling undertaken in domestic settings where earthen floors are common suggests that floors can be highly contaminated with faecal indicator markers as well as with specific disease-causing pathogens.^12-18^

In-depth observational studies mapping child behaviour and routines within their homes have shown that infants and children under the age of 5 face considerable exposure to household floors.^19^ Children often explore environments, including floors, with their hands, frequently perform hand-to-mouth actions, and can engage in geophagia.^19-21^ Compounding this heightened risk of exposure is the increased vulnerability that children have towards many enteric infections, which are far more likely to cause severe-morbidity and mortality than in adults.^3^

Previous studies have identified type of floor (earthen vs improved), sanitation access, distance to water source, and presence of animals to be associated with levels of enteric pathogen contamination on household floors.^13,22,23^ However, little work has been done to investigate the pathways through which these factors, and other possible determinants, lead to contamination of domestic floors with human and animal faeces. Identifying these pathways will allow for a clearer understanding of how household flooring contributes to enteric and parasitic disease transmission in the domestic setting and help identify scenarios in which household flooring should be considered an important domain of transmission.

The SABABU (*Sakafu Bora Afya Bora – Utafiti)* project is a three-yearstudy investigating the relationship between household flooring and human health and wellbeing. The study involves implementing a cluster-randomised controlled trial investigating the impact of an improved household flooring intervention on enteric and parasitic infections among children and caregivers in groups of villages in Kenya. To design the trial’s intervention, we conducted formative research in eligible villages in Kwale, Bungoma and Narok counties to explore current housing conditions, household daily routines, and community perspectives on domestic hygiene issues. The analysis described in this paper uses data from this formative research period including^1^: household censuses that collected quantitative data on housing conditions, WASH access, socioeconomic indicators and demographics^2^; unstructured observations of household daily routines; and^3^ in-depth interviews (IDIs) carried out with primary caregivers. The objective of this paper is to hypothesise the pathways through which floors become contaminated with faecal matter in 3 culturally and environmentally diverse settings in rural Kenya. Understanding the factors that may impact faecal contamination of domestic floors in these settings will provide insights that could be of value to future housing, WASH, and One Health interventions aiming to limit human contact with harmful pathogens in the domestic environment. It should be noted that environmental sampling was outside of the scope of this formative work, as such the objectives of this study are hypothesis generating.

## Methods

### Ethics statement

Ethical approval for this study was obtained from the Kenya Medical Research Institute (KEMRI) Scientific and Ethics Review Committee (SERU No.4157) and the London School of Hygiene & Tropical Medicine (LSHTM) Ethics Committee (22916). For households participating in the census written informed consent was provided by an adult household member and was recorded using an electronic signature traced directly on to a mobile device as well as on a paper form and information sheet that was retained by the household. For participants in household observations written informed consent (or written or verbal assent for children) was obtained from all participants.

### Study setting

**Figure 1.**
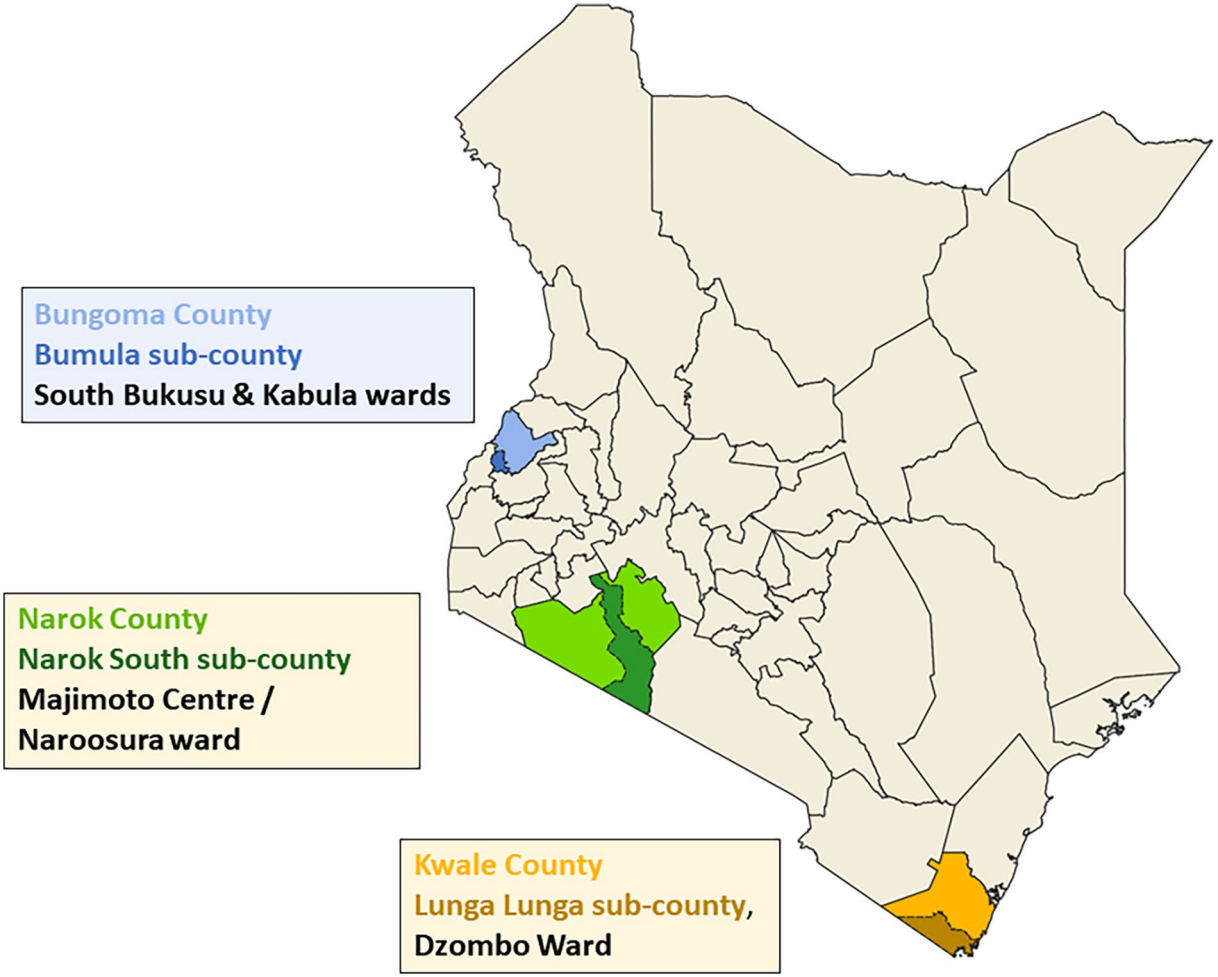
Map of study settings.

This study took place in 3 counties in Kenya (Kwale, Bungoma and Narok) between the months of May and October 2021 (Figure 1). In Kwale, the study was conducted in 7 contiguous villages in Dzombo ward, Lunga Lunga sub-county. The climate in Dzombo is warm semi-humid with 2 distinct rainy seasons (March-May and September-November), and the primary occupation is subsistence farming. In Bungoma county, data was collected in 5 villages in the South Bukusu ward and 2 villages in Kabula ward within Bumila sub-county where the climate is cool humid and rainfall is experienced throughout the year and the primary occupation is also subsistence farming. In Narok county, data was collected in 10 villages in the Majimoto Centre/Naroosura where communities are mostly pastoralists. The climate in Narok is semi-arid, with the driest months occurring between June and October.^24^ Prevalence of childhood diarrhoea in Narok and Bungoma (17% and 18% respectively) were slightly above the national average of 14%, while in Kwale prevalence was well below at 4%.^25^

### Data collection

A census of all households was first conducted in study villages to record population demographics, building characteristics, levels and quality of WASH access and use, and animal ownership data. Data were recorded electronically using Android phones preloaded with SurveyCTO software (Dobility, Inc; Cambridge, MA, USA and Ahmedabad, India). Data were collected through a combination of self-reported measures (household demographic data, animal ownership, asset ownership, and some WASH measures) and direct observation (building characteristics and some WASH indicators). Trained field officers administered the questionnaire and were accompanied by local guides (either community health volunteers or village elders). Completeness was validated using community guides’ knowledge of household locations and overlaying household GPS coordinates on to satellite imagery to identify unvisited structures. A household was defined as a family that shares the same cooking pot and that lives and eats together. The building(s) and outside area that households occupy were collectively referred to as household compounds.

Following completion of the census, unstructured observations were conducted in a stratified random sample drawn from censused households that had a child under the age of 5. Eighteen households were selected each in Bungoma and Kwale and 16 households in Narok (total n = 52) (Table 1). In Bungoma and Kwale sampling was stratified by animal ownership (owned poultry or livestock vs owned no animals) and type of floor present (earthen vs improved), and in Narok sampling was stratified by type of household (single household vs household located in compound with other related households) and also by type of floor. Stratification groupings differed between Bungoma/Kwale and Narok because in Narok, the location of households inside *manyatta* compounds (a group of multiple, usually related, households congregated within a single demarcated boundary) was very common and was expected to play a significant role in how animal husbandry practices were conducted.

**Table 1.** Household sampling for observations and IDIs.

Household sampling group	Bungoma	Kwale	Narok
Unimproved floor and own animals	6	6	NA
Unimproved floor and do not own animals	6	6	NA
Improved floor with or without animals	6	6	NA
Unimproved floor not located in a compound	NA	NA	5
Unimproved floor located in a compound	NA	NA	9
Improved floor located either in compound or as single household	NA	NA	2

In advance of the observations, field officers visited sampled households to obtain informed consent from all present household members and to create a floorplan of the compound, which provided a visual context within which to analyse field notes (Document S1).

During observations, field officers passively observed daily routines with a focus on pre-identified activities of interest that were expected to capture the core components of household members daily routines. These included floor cleaning activities, animal husbandry practices, caregiving, food preparation, meal preparation and eating, water storage and collection, personal hygiene behaviours, laundry, and sleeping arrangements. Observations occurred over a 2-day period with 2 sessions per day; 1 in the morning (7-10am) and the second in the afternoon (12-3pm). Sessions were scheduled to target the busy morning and lunchtime routines (Document S2). Field officers employed real-time note taking and concurrent video-recorded footage to capture routines. Video recording was done passively by placing a movement-activated camera in the ‘busy’ room of the household or in the kitchen. Cameras were only installed with the express verbal and written consent of household members. Notes were reviewed and synthesised into summaries by field officers following completion of the observations (Document S3). Video footage was watched by investigators and activity-ordered summaries (eg, food preparation, child caregiving, animal husbandry) were produced (Document S4).

To provide additional context and to explore the motivations behind the observed routines, IDIs were conducted with caregivers in the same households where observations were undertaken (Document S5). Interviews were audio recorded and transcribed into English prior to coding. For the purpose of this analysis, ‘interior floors’ refer to floors inside household buildings and ‘exterior floors’ refer to the ground outside that is within a household’s compound (eg, the courtyard area). References to ‘improved floors’ indicate the presence of cement, concrete or ceramic tiled floors that are sealed and washable.

### Data analysis

Management and analysis of census data was done using R (version 4.1.3)^26,27^ and STATA 16.0 (Stata Corp, College Station, Texas). Following data assembly and cleaning, education and asset indexes (not including floor type or other housing indicators) were developed for each study setting as a proxy measure for socio-economic status. To do this an iterative principal component analysis (PCA) of binary asset and education variables was completed (Table S1). Items were retained in the final PCA if they had an item-rest correlation of >.1 and the PCA was considered to have acceptable levels of internal consistency when the overall alpha was >.7.^28^ Households were then categorised into quintiles based on index scores and graphed against determinants of interest using bar charts and box plots. Associations between asset and education index score and outcomes of interest (namely household floor type and self-reported sharing of rooms between animals and household members) were estimated using multilevel logistic regression models outputting odds ratios (ORs) and 95% confidence intervals (95% CIs), with random intercepts to account for nesting of households within villages. Models were adjusted for number of buildings in the household compound.

Data from observations and IDIs were analysed thematically with NVivo 12 (QSR International Pty Ltd, 2018) – using key activities of interest as the starting point for development of key codes and themes. Pre-defined activities included food preparation, floor hygiene, child caregiving, animal husbandry, personal hygiene, water collection, use, and storage, and laundry. Observation summaries, video summaries, household floorplans, and transcripts from interviews were used by investigators to produce triangulated household-level case-memos that documented when, where, how, and by whom each routine activity was practiced (Document S6). Household case memos were then grouped by stratification and study setting and used to produce top-level activity summaries. Activity summaries were reviewed and compared between stratification and study site groups to identify points of concurrence and divergence between groups of interest.

After routines were defined, results between and within groups were further analysed to explore possible determinants of contamination of floors and the relationships among those determinants. Following this analysis, a conceptual framework was developed to map the interrelatedness of determinants. Determinants were categorised as either being proximal (directly facilitating the contamination of domestic floors with faecal matter) or distal (facilitating contamination of domestic floors through proximal determinants).

## Results

### Community characteristics

Totals of 906, 812, 1102 households were recorded in the study census in the Bungoma, Kwale and Narok study settings, respectively. Observations and interviews were conducted in a sub-sample of 18 households each in Kwale and Bungoma and in 16 households in Narok. While all settings were rural and comprised in the majority by households with earthen floors (75%, 75%, 82% respectively of censused households in Kwale, Bungoma and Narok (Document S7)) notable differences in how homes were organised and how and which animals were owned by households were revealed by the census, observations and interviews.

In the Kwale and Bungoma communities, households tended to occupy multiple buildings with 73% (Kwale) and 70% (Bungoma) of homes recorded in the census having at least 2 buildings. While these additional buildings served as kitchens, bedrooms, or store rooms, many activities such as certain aspects of food preparation were seen during observations to be undertaken outside in household courtyards. Access to sanitation and nearby water sources was high in both Kwale and Bungoma, with 89% (Kwale) and 97% (Bungoma) reporting access to at least limited sanitation and 83% (Kwale) and 86% (Bungoma) being within a 30-minute round trip from their primary water source. In Narok, the census showed that the majority of homes were comprised of 1 building (74%) while observations revealed that families conducted daily activities such as food preparation, eating, and dish washing inside, so as to avoid dust in the environment. WASH access was more limited than in Kwale and Bungoma, with 19% households reporting access to at least limited sanitation and 66% being within a 30-minute round trip from their primary water source. Animal ownership was high in all settings, however the types of animals owned varied. While chickens and other poultry were the most commonly owned animals in Kwale (72% households owning poultry) and Bungoma (73% households owning poultry), cattle and goats were the most commonly owned animals in Narok (70% households owning cattle and 72% of households owning goats) – where pastoralism is the primary income generating activity.

This observed diversity between study settings in building culture, animal ownership, and use of space within the home allowed us to hypothesise a set of common determinants for floor faecal contamination and explore their interrelatedness. Based on these determinants we developed a generic conceptual framework for floor faecal contamination which we present below along with our findings which underpin each determinant.

### A conceptual framework for determinants of floor faecal contamination

**Figure 2.**
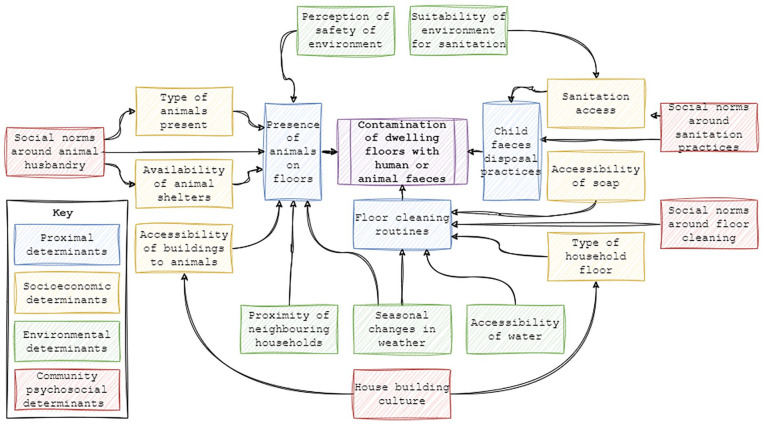
Conceptual framework hypothesising the proximal and distal determinants of floor faecal contamination in villages in Bungoma, Kwale and Narok counties, Kenya.

Analysis of observation and interview data revealed 3 key potential proximal determinants for faecal contamination of domestic floors; presence of animals on floors; child faeces disposal practices, and; floor cleaning activities. In addition to these, 3 categories of distal determinants were also hypothesised; socioeconomic; environmental, and; community psychosocial (Figure 2 and Table 2).

**Table 2.** Proximal and distal determinants of floor faecal contamination in villages in Bungoma, Kwale, and Narok counties.

Proximal determinants	Socioeconomic distal determinants	Environmental distal determinants	Community psychosocial determinants
Presence of animals on floors – this causes contamination through defecation directly onto floors or transporting faecal matter on their bodies	Type and quantities of animals present – poultry more likely to gain access to interior spaces than goats or cattle.Dedicated animals shelters reduce need to house animals in kitchens and other spaces shared with household members.Accessibility of interior spaces – open doors and holes in walls allow chickens and goats to access interior floors.	Perceptions of safety of environment – cold weather, fear over predators or thieves encourages household to house animals in living rooms or kitchens.Close proximity of households in a community allows animals to access neighbours’ compounds.Seasonal changes in weather determine animals daily routines and where they are housed during the night.	Cultural preferences influence which animal types are owned and how they are housed.House building culture influences whether the buildings have holes in walls that allow animals access to interior floors.
Child faeces – comes into contact with floor through infants and young children defecating directly on to floors or cleaning materials being deposited on floor	Presence of sanitation on compound allows for disposal of child faeces, but does not prevent initial contact with floors.	A high water table or soil types with high sand content. may preclude construction of latrines.	Social norms around sanitation practices and child faeces disposal.
Floor cleaning – sweeping or mopping with water and soap removes faecal matter from interior and exterior floors	Presence of an improved floor allows households to incorporate mopping with water and soap into their routines.Access to soap can make floor cleaning more effective at removing faecal matter.	Seasonal changes in weather can effect amount of dirt being brought on to household floors and thus the amount of cleaning required.In water-scarce settings households may not have sufficient access to water to practice regular mopping.	Cultural norms around what constitutes ‘regular’ cleaning.House building culture determines the desirability of improved floors.

### Proximal determinants – presence of animals on floors

Animal presence on floors (primarily poultry, goats, and cattle in these settings) was identified as a potential proximal determinant of faecal contamination as they can either defecate directly on to floors, or bring faecal matter on to the floor on their body. Among households participating in the observations and IDIs, animals were observed entering inside household buildings frequently as well as spending time on exterior floors in household courtyards. However, the type of animal present, decision on where to house animals during the night, animal routines during the day, and the building culture of a community were all observed to modulate the level of exposure that animals had to interior and exterior floors (Table 3).

**Table 3.** Summary of animal exposure to floors by animal type.

Animal type	Nighttime exposure to floors	Daytime exposure to floors
Chickens and poultry	In Kwale and Bungoma chickens were often housed at night in rooms that were used for other purposes during the day, for example kitchens; greatly increasing their contact with interior floors.	Chickens had considerable exposure to exterior courtyard floors as they were free to roam during the day – In Kwale and Bungoma they were also often observed gaining access inside household buildings and thus increasing their contact with interior floors. In Narok, this was less common.
	In Narok chickens were more likely to have dedicated shelters; reducing their contact with interior floors.	
Goats	A lot of variation was observed between and within study settings. Goats were housed variously in duel-purpose rooms, in dedicated shelters, and sometimes outside without shelter.	A lot of variation was observed between and within study settings. Goats were sometimes left to roam in and around the compound, sometimes tethered close to courtyard floor, and sometimes (always in Narok) taken off the household compound to graze elsewhere.
Cattle	Housed in dedicated shelters or sheds within the household compound in all three sites. During the dry season in Narok, they are resettled away from the household.	Usually taken off the household compound during the day to be grazed elsewhere.

**Figure 3.**
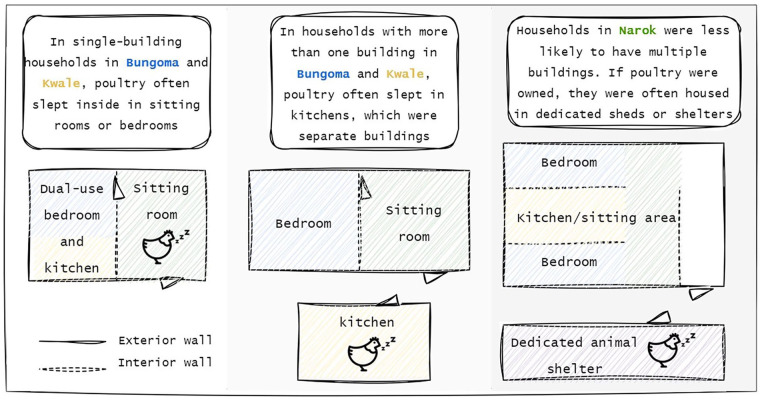
Example household layouts in Bungoma, Kwale and Narok and how these influenced where poultry were housed.

Of notable importance was the type of animal present in the home. In all 3 study settings, in households where cattle were owned and kept, they were almost always housed at night in dedicated sheds or more permanent structures that served a single purpose as an animal shelter, thereby keeping them away from contact with the interior floors used by household members for daily activities such as food preparation, dish washing, and caregiving. Whereas for goats, chickens and other poultry, arrangements were more varied. The majority of observed households in the Kwale and Bungoma communities that owned chickens or other poultry housed them in rooms that had dual purposes, for example serving as a living room during the day and then as an animal shelter during the night. Among single-building households, chickens slept inside, sometimes in the same room as household members, greatly increasing their contact with interior floors (Figure 3). There was considerable variation in how goats were sheltered during the night. Some households kept them in dual-purpose rooms, such as kitchens, some kept them dedicated shelters, such as animal sheds, and some let them sleep outside without shelter. There was no indication from interview respondents in Kwale and Bungoma that animal sleeping arrangements had seasonal variations. However, in Narok, during the dry season, cattle and goats were reported by some household to be taken further from the household in search of pastures and during the rainy season, young goats or cattle may be kept inside during the night to protect them from the elements.

[caring for the cattle] changes because during the dry season we will resettle them to a different [place] and during rainy season we will bring them together at home and look out for them.

*Primary caregiver, Narok*

Daytime routines were also different depending on the animal type. Cattle were typically taken to graze outside of the compound during the day so they spent only a short period of time on exterior courtyard floors. In some households, cattle were observed to be tethered and left to graze in the vicinity of the compound – thereby having some contact with exterior courtyard floors. However, in the majority of households, while in the compound, cattle were usually contained within their shed or shelter with only limited contact with exterior floors, and in no instance were any cattle observed entering inside household buildings. Goats, while sometimes taken off-compound to graze with the cattle (particularly in Narok), had more contact with the exterior ground and interior floors. In many instances they were tethered and left to graze in and around household compounds during the day. Goats in Bungoma were observed entering into household buildings during the day.

In all households that owned chickens, they roamed during the day to find food. In all 3 settings, this meant that chickens spent considerable amounts of time on the exterior courtyard ground. In Kwale and Bungoma chickens were frequently observed entering inside buildings throughout observation periods using open doors or gaps in the wall – greatly increasing their exposure to interior floors. Chicken and poultry contact was not limited to households that owned animals; neighbours’ animals were frequently observed entering the compounds of these households. For both chickens and goats during the daytime, the impression from observers was that these animals were drawn towards parts of the compound where food was being stored, prepared, cooked, eaten, or utensils had been washed. Narok was the exception, where both chickens and goats were only infrequently observed entering inside buildings. Possibly due to the fact that cooking and food storage areas were located deeper inside buildings and thus harder for chickens and goats to reach.

### Proximal determinants – child faeces disposal practices

Children’s defecation practices and the disposal of their faeces were observed to be an important potential pathway through which faecal matter came into contact with floors. In Kwale and Bungoma, where sanitation access is high, pre-school aged children would defecate outside but within the compound, with the faeces then deposited in the household latrine by an elder household member. Water used to clean the child would be deposited either on the courtyard ground or outside of the compound. In Narok, where sanitation access was much less prevalent, children’s faeces was observed being deposited by adult household members in the field outside of the compound. Thus, in these settings, sanitation access did not prevent child faeces from coming into contact with interior or exterior floors, but it is likely effective in preventing it from being deposited in the wider environment.

### Proximal determinants – floor cleaning routines

Floor cleaning activities such as sweeping and mopping were observed as the primary method for removing faecal matter from floors. Activities were embedded within daily routines in all 3 study settings, with cleaning often taking place early in the morning and occasionally after lunch or in the evening. Regular sweeping was considered to be a ‘normal’ part of the daily routine by respondents. The reason most often cited was to remove dirt, and to reduce bad smells.

‘Dirt [prompts me to clean the floor] . . .It is normal for a house to be swept every day. Then these chicken sleep at the front so I must clean it daily because of the smell’.

*Primary caregiver, Kwale*

The type of floor present in the household strongly influenced what floor cleaning activities were carried out. Earthen floors (including both interior and the exterior courtyard ground) had dried debris removed with metal hoes (*jembes*), were swept with reed or twig brooms, and occasionally had water sprinkled either before or after sweeping to keep dust levels in-check. Debris from floors was either deposited in compost heaps within the compound or around the edge of the courtyard area. Improved floors tended to have more extensive cleaning regimens, with floors being swept first and then mopped – sometimes with soap and water and sometimes solely with water. As a result households could more effectively remove debris and faecal matter from these floors. However, wastewater was most often deposited directly on to the exterior courtyard floor – possibly moving faecal matter from interior to exterior floors. Seasonal changes in weather were reported as impacting floor cleaning routines as the arrival of the rains increased the amount of mud in the house which can necessitate more frequent cleaning.

‘Lets say like right now it is muddy, I clean up to 3 times [in a day] and then when we have the sunny season, I may clean once a day or not, I just sweep, [and] the floor will still be clean’.

*Primary caregiver, Bungoma*

### Distal determinants – socio-economic factors

**Figure 4.**
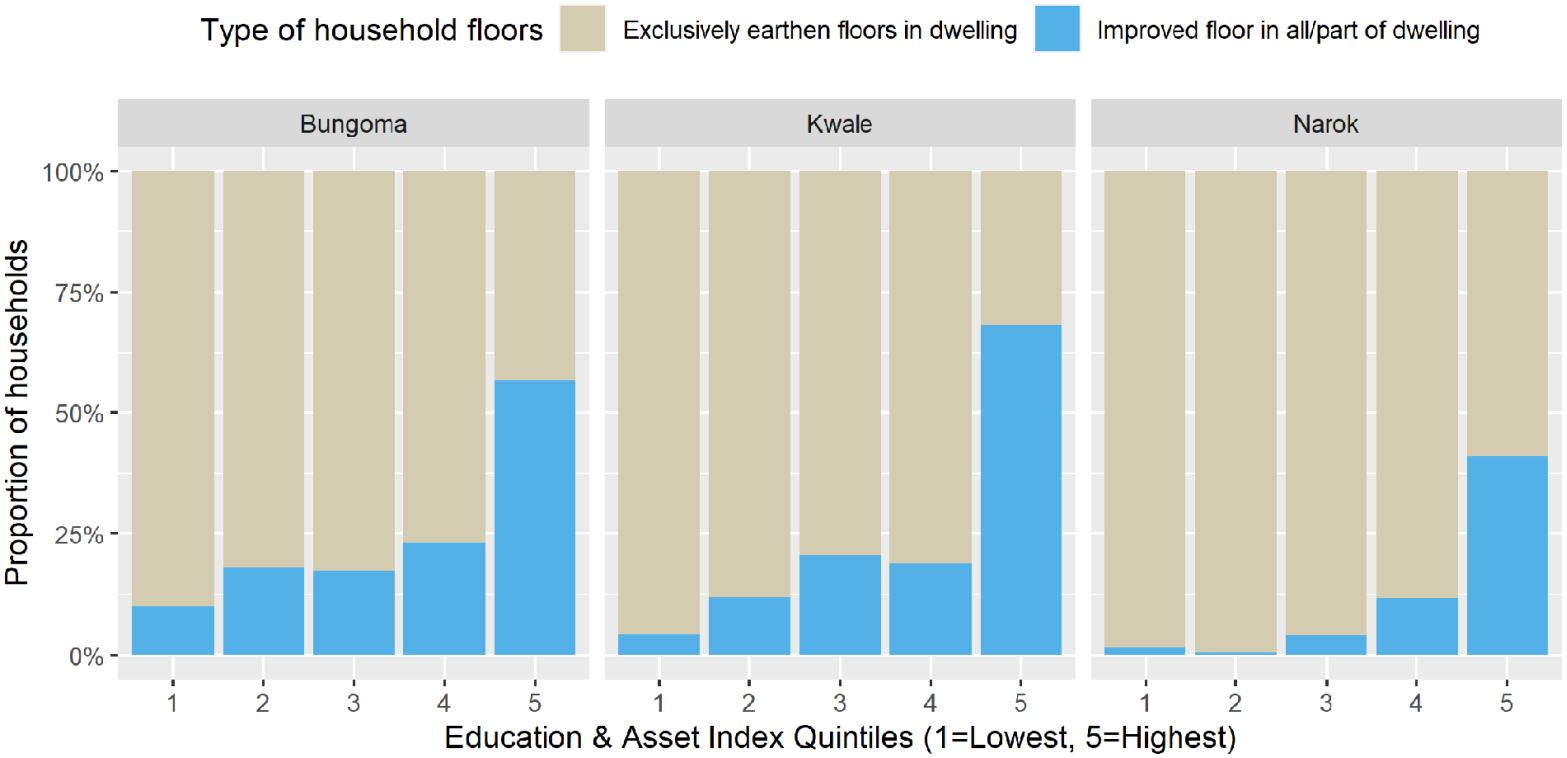
Proportion of censused households in each study settings with at least 1 improved floor by education and asset index groups.

The socio-economic status (SES) of households was hypothesised as a distal determinant of floor faecal contamination. As identified above, households with improved floors practiced enhanced floor cleaning practices such as mopping – which likely decreased floor faecal contamination. In all 3 settings, census data showed that households with higher education and asset index scores were more likely to have at least 1 building with an improved floor (Table 4 and Figure 4).

**Table 4.** Association between ownership of an improved floor and educational and asset index score.

Variable	Bungoma		Kwale		Narok	
	HHs with improved floor, n (%)	Adjusted odds ratio (95% CI)^a^	HHs with improved floor, n (%)	Adjusted odds ratio (95% CI)^a^	HHs with improved floor, n (%)	Adjusted odds ratio (95% CI)^a^
Education and asset index quintile						
Lowest	18 (9.9)	1 (ref)	7 (4.2)	1 (ref)	4 (1.6)	1 (ref)
Second	33 (18)	1.74 (0.92-3.28)	18 (11.9)	2.89 (1.16-7.2)	1 (0.5)	0.32 (0.04-2.9)
Third	31 (17.3)	1.51 (0.79-2.86)	35 (20.6)	5.42 (2.3-12.8)	9 (4.1)	2.58 (0.77-8.62)
Fourth	42 (23.1)	1.93 (1.04-3.6)	30 (18.9)	4.9 (2.04-11.73)	26 (11.8)	7.6 (2.54-22.75)
Highest	102 (56.7)	8.05 (4.45-14.56)	114 (68.3)	45.12 (19.29-105.55)	90 (41.1)	40.82 (14.24-117.05)

a Adjusted by village and number of buildings in the compound.

**Figure 5.**
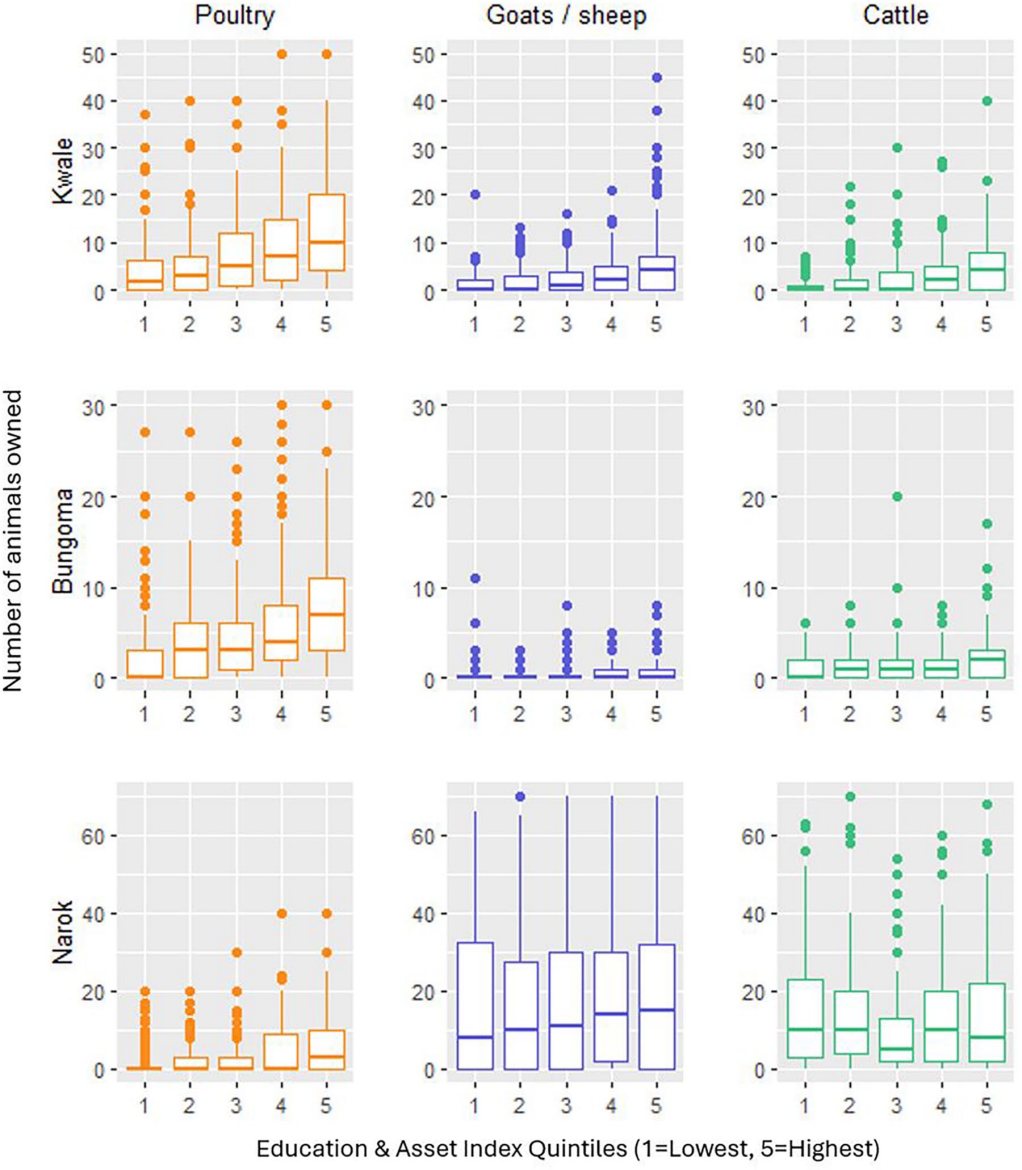
Animal ownership by education and asset index quintiles in each study setting.

There was a complex relationship between SES and animal ownership, identified above as a proximal determinant of floor faecal contamination. In Bungoma, there was a positive association between the number of poultry owned and the education and asset index scores of households, with higher scoring households more likely to own more poultry and therefore potentially having floors exposed to greater animal activity (Figure 5). In Kwale, the same trend was observed in poultry ownership, as well as with cattle and goats /sheep. In Narok there was not a clear relationship between the educational and asset index score of households and the number of cattle or goats/sheep owned. This reflects an important contextual reality observed in Narok, where wealth (In the setting of Narok, normally denoted by numbers of livestock owned) did not always correlate with asset ownership, floor type, or educational attendance.

**Figure 6.**
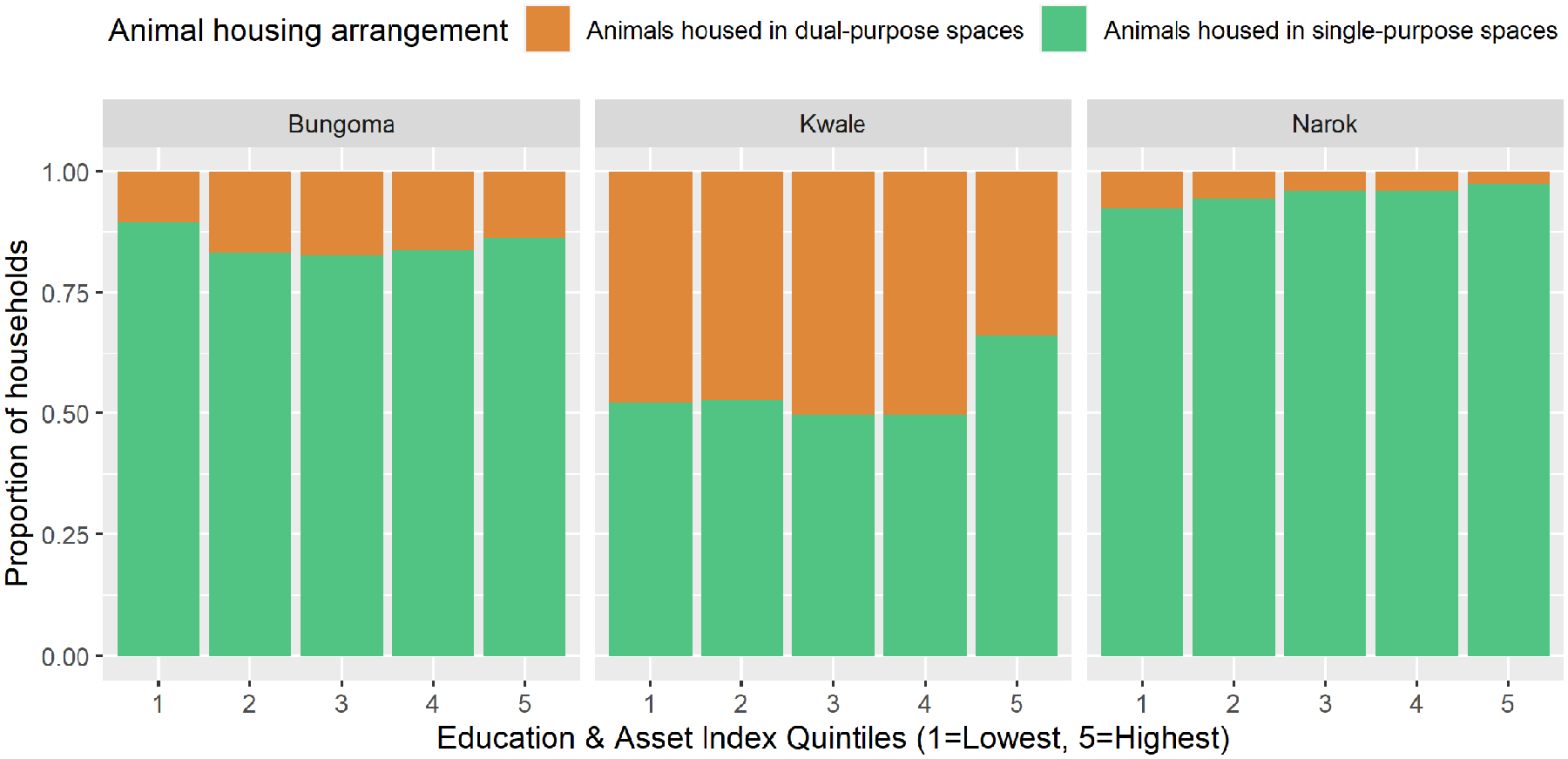
Animal housing arrangements by education and asset index quintiles in each study setting.

As identified above, animals that were housed in dedicated sheds during the night had far less contact with interior floors, when compared with animals that slept in rooms with dual-purposes (eg, a room serving as kitchen during the day and then as an animal shelter during the night). In Kwale, there was some indication that SES was associated with the presence of dedicated animal shelters, with households in the highest education and asset index quintile being significantly less likely to house animals in dual-purpose rooms that are used by household members for other activities compared with households in the lowest quintile, thereby reducing animal exposure to parts of the compound used by household members (Figure 6 and Table 5). In Narok, data from the census showed that shared spaces between animals and household members were extremely rare (Figure 6 and Table 5) despite far greater numbers of cattle and goats/sheep being owned compared with the other 2 settings. In Bungoma data from the census indicated that only 15% of households reported shared spaces between animal and household members (although it was more common than in Narok where only 5% reported shared spaces) and that there didn’t appear to be a relationship between education and asset index score and animal housing arrangements (Table 5). However, 2 households in Bungoma that participated in the observations and IDIs explained that the chickens slept in the same room as household members because they had not yet built a dedicated shelter for them.

**Table 5.** Association between households housing animals in dual-purpose rooms versus dedicated rooms/shelters and household educational and asset index score.

Variable	Bungoma		Kwale		Narok	
	Animals in dual-purpose spaces, n (%)	Adjusted odds ratio (95% CI)^a^	Animals in dual-purpose spaces, n (%)	Adjusted odds ratio (95% CI)^a^	Animals in dual-purpose spaces, n (%)	Adjusted odds ratio (95% CI)^a^
Education and asset index quintile						
Lowest	10 (10.4)	1 (ref)	57 (47.9)	1 (ref)	14 (7.5)	1 (ref)
Second	24 (16.8)	1.78 (0.79-4.03)	61 (47.3)	0.92 (0.55-1.55)	8 (5.6)	0.72 (0.28-1.85)
Third	27 (17.4)	1.82 (0.82-4.06)	76 (50.3)	1.04 (0.63-1.72)	7 (3.9)	0.49 (0.18-1.32)
Fourth	27 (16.3)	1.88 (0.84-4.21)	72 (50.3)	1.08 (0.65-1.8)	8 (3.9)	0.45 (0.17-1.22)
Highest	23 (13.7)	1.46 (0.64-3.35)	51 (33.8)	0.52 (0.31-0.88)	5 (2.6)	0.29 (0.09-0.9)

a Adjusted by village and number of buildings in the compound.

### Distal determinants – community psychosocial factors

The unique cultural context of each setting was also observed to influence the hypothesised proximal determinants of floor faecal contamination. Norms around animal ownership and husbandry habits differed considerably between the 3 study settings. In Narok, while overall animal ownership was higher when compared with Bungoma and Kwale, housing animals in dual-purpose spaces, such as kitchens, was a far less common practice, with most animals housed either off compound or in dedicated sheds, pens or rooms. In Bungoma, there was a strong theme of households preferring to house chickens close to where household members sleep to ensure their security.

‘[Chickens sleep inside] Because of cold and also so that thieves don’t steal, also chicken can’t just sleep outside’

*Primary caregiver, Bungoma*

Building culture also appeared to play an important role in influencing floor contamination, with study communities in Narok, less likely to have improved floors. Observations also revealed significant variations in types of earthen floors between study settings. While in Kwale, earthen floors were typically uneven and without any finishing layer applied, in Bungoma and Narok, many earthen floors had a finish comprised of animal dung, ash and clay that provided a semi-sealed finish, that could be more easily swept.

### Distal determinants – environmental factors

The environmental and climactic conditions that households are located within was also observed to impact hypothesised proximal determinants of floor faecal contamination. Households located in communities in Bungoma and Kwale with close proximity between compounds often had animals from neighbouring households roaming into their compounds, indicating that lack of ownership of animals does not protect floors from becoming contaminated by them. In Narok, where compounds were more spread out (outside of manyatta compounds), this was not observed. The greatest reported seasonal variation in animal husbandry practices was in Narok where during the dry season, cattle and goats were reported by some household to be taken further from the household in search of pastures. In addition to this during the rainy season, young goats or cattle may be kept inside during the night to protect them from the elements.

The broader perception of the lived environment and its risks to the safety of animals also affected where they were housed. Specifically, perceptions around risk of theft, predators, and adverse weather were also found to be important determinants of where households chose to house animals – with many respondents in IDIs stating that chickens particularly would be killed or stolen if left outside during the night.

‘The chickens sleep in the [main] building because of thieves and also there are animals that can come at night to disturb the chickens so you have to have them close’.

*Primary caregiver, Bungoma*

## Discussion

In this paper we hypothesised 3 proximal determinants of floor faecal contamination – presence of animals within the compound, child faeces disposal, and floor cleaning practices. Beyond this, we showed that the relative importance of these proximal determinants is influenced by a range of socio-economic, environmental, and community psychosocial factors. Presence of animals on household floors was affected by the type of animals owned – with poultry more likely to gain access to interior spaces than goats or cattle, the use of dedicated animal sheds which reduce the need to house animals in kitchens or other shared spaces at night, the ability of animals to access neighbouring compounds, and the building culture present in a community – which can determine the accessibility of interior spaces to animals. Effective floor cleaning practices, namely mopping with soap, were dictated by the presence of an improved floor which in Kwale and Bungoma was associated with socioeconomic status. Child faeces was seen coming into contact with interior and exterior floors regardless of whether or not households had access to sanitation. Through these findings we argue that the relative contribution of these proximal determinants depends on the socioeconomic, environmental, and cultural context in which they occur. In our study, variations in these factors meant that the setting with highest level of animal ownership (Narok) saw the least level of contact between animals and interior floors, while 2 settings with high levels of sanitation access (Kwale and Bungoma) saw similar levels of contact between household floors and child faeces as the setting with extremely low access to sanitation (Narok).

Studies in India and Bangladesh have identified animal ownership as an important risk factor for the presence of enteric pathogens in the domestic environment.^13,29^ Results from this study add insight to these findings, identifying the conditions under which animal contact with domestic floors are more likely, and thus the risks to human health increased. The type of animal owned, whether they are housed in shared-purpose rooms (ie, kitchens or bedrooms), and the extent of animal roaming during the day were all noted in our study to be important determinants of animal contact with human floors. This is supported by a previous study from Ethiopia investigating the transmission pathways for *Campylobacter* spp. in the domestic environment that found that keeping animals inside during the day was a risk factor for floor contamination, with the authors suggesting the prevailing practice of housing animals in shared-purpose spaces was the reason for this association.^23^ These findings also have relevance for the transmission of soil-transmitted helminths as previous studies have identified helminth eggs in pigs, goats, chickens, and dogs, and have linked their presence to environmental contamination in domestic settings.^30,31^

The relationship between socio-economic status and floor faecal contamination has rarely been directly explored, with studies usually investigating its relationship with health outcomes such as diarrhoea or enteric infections. A study in Peru found that monthly income (recorded as a proxy for SES) was not associated with levels of *E. coli* on household floors.^22^ Results from our study suggest there may be an important but complicated relationship between SES and floor faecal contamination. Higher scores in education and asset indices were associated with increased odds of having an improved floor in all 3 study settings, indicating that higher SES may reduce floor faecal contamination through influencing the type of floors present in the household. That the most notable increases in improved floor ownership only appeared in the highest wealth quintiles in each study setting indicate the high relative cost of improved flooring and potentially the lower prioritisation attached to it by households. However, higher scores in the education and asset index in Bungoma and Kwale were also associated with increased animal ownership, indicating that higher SES may result in increased levels of animal contact with household floors. In Narok, education and asset index scores were detached from animal ownership, suggesting that wealth (represented by the number of animals owned) did not always correlate with higher levels of education and asset ownership, a phenomenon noted by Randall^32^ in their study of the demographics of migratory populations. Finally, there was some indication that SES affected how households housed their animals in Kwale, with higher education and asset index scores being associated with a higher chance of animals being housed in dedicated shelters, away from contact with the interior household floors.

In this study, sweeping of floors was observed to be practiced on a daily basis by almost all households across all 3 study settings, regardless of the type of household floors present. Households with improved floors were almost all observed to incorporate mopping into their floor hygiene routines. This suggests that the barriers to households practicing effective floor hygiene are primarily technological and based on the fact that earthen floors cannot be mopped. Future household flooring interventions may be able to achieve effective floor hygiene practices simply by providing the technology (ie, an improved floor) and without allocating significant resources to behaviour change activities.

Our observation that household access to sanitation may not prevent human faeces from coming into the contact with household floors due to child faeces disposal practices supports findings from studies in Bangladesh and Ethiopia which found no relationship between sanitation access and the level of human faecal markers on household floors.^23,33,34^ A study in India also found that sanitation access was no guarantee of safe disposal of child faeces.^35^ However, a study in Peru found a significant relationship between levels of *E. coli* on household floors and sanitation type and sharing status,^22^ suggesting that the role of sanitation access in determining floor faecal contamination may be contextually sensitive.

There are some limitations with this research. Notably, this study did not include environmental sampling of household floors and as such we are not able to present data on the presence of either faecal indicator bacteria or specific pathogens on household floors. However, through observations, interviews, and censuses, we believe this study is well placed to describe pathways through which human and animal faeces come into contact with domestic floors. Another limitation is that observations were undertaken in each site at only one timepoint in the year, so it is possible that some seasonal variations in behaviours were missed. To address this, we included questions on seasonal variations in routines within IDI question guides.

Enteric and parasitic infections such as soil-transmitted helminths continue to cause considerable morbidity and mortality among infants and children and household floors are increasingly being understood as an important domain of transmission for these infections.^36,37^ In this study we identified proximal determinants and upstream factors that may be driving floor faecal contamination in 3 settings in rural Kenya. Understanding the pathways through which household floors come into contact with faecal matter is an important foundational step towards designing effective interventions to reduce household floor contamination and thus transmission of these infections. The findings from this study could have application for a wide range of interventions that aim to reduce human contact with enteric pathogens in the domestic environment including sanitation, animal corralling, child faeces disposal, and household flooring interventions. Future studies should look to combine observations of household routines with environmental sampling that can identify the presence of faecal indicator bacteria or known pathogens of interest.

## Supplemental Material

sj-docx-1-ehi-10.1177_11786302241246454 – Supplemental material for Identifying Potential Determinants of Faecal Contamination on Domestic Floors in Three Settings in Rural Kenya: A Mixed Methods Analysis Supplemental material, sj-docx-1-ehi-10.1177_11786302241246454 for Identifying Potential Determinants of Faecal Contamination on Domestic Floors in Three Settings in Rural Kenya: A Mixed Methods Analysis by Hugo Legge, Karisa Kazungo, Sharon Muli, Lynne Elson, Jacinta Mwongeli, Katherine E Halliday, Victoria Ochwal, William Oswald, Robert Dreibelbis, Doris Njomo, Charles Mwandawiro, Ulrike Fillinger, Rachel Pullan and Stella Kepha in Environmental Health Insights

sj-docx-2-ehi-10.1177_11786302241246454 – Supplemental material for Identifying Potential Determinants of Faecal Contamination on Domestic Floors in Three Settings in Rural Kenya: A Mixed Methods Analysis Supplemental material, sj-docx-2-ehi-10.1177_11786302241246454 for Identifying Potential Determinants of Faecal Contamination on Domestic Floors in Three Settings in Rural Kenya: A Mixed Methods Analysis by Hugo Legge, Karisa Kazungo, Sharon Muli, Lynne Elson, Jacinta Mwongeli, Katherine E Halliday, Victoria Ochwal, William Oswald, Robert Dreibelbis, Doris Njomo, Charles Mwandawiro, Ulrike Fillinger, Rachel Pullan and Stella Kepha in Environmental Health Insights

sj-docx-3-ehi-10.1177_11786302241246454 – Supplemental material for Identifying Potential Determinants of Faecal Contamination on Domestic Floors in Three Settings in Rural Kenya: A Mixed Methods Analysis Supplemental material, sj-docx-3-ehi-10.1177_11786302241246454 for Identifying Potential Determinants of Faecal Contamination on Domestic Floors in Three Settings in Rural Kenya: A Mixed Methods Analysis by Hugo Legge, Karisa Kazungo, Sharon Muli, Lynne Elson, Jacinta Mwongeli, Katherine E Halliday, Victoria Ochwal, William Oswald, Robert Dreibelbis, Doris Njomo, Charles Mwandawiro, Ulrike Fillinger, Rachel Pullan and Stella Kepha in Environmental Health Insights

sj-docx-4-ehi-10.1177_11786302241246454 – Supplemental material for Identifying Potential Determinants of Faecal Contamination on Domestic Floors in Three Settings in Rural Kenya: A Mixed Methods Analysis Supplemental material, sj-docx-4-ehi-10.1177_11786302241246454 for Identifying Potential Determinants of Faecal Contamination on Domestic Floors in Three Settings in Rural Kenya: A Mixed Methods Analysis by Hugo Legge, Karisa Kazungo, Sharon Muli, Lynne Elson, Jacinta Mwongeli, Katherine E Halliday, Victoria Ochwal, William Oswald, Robert Dreibelbis, Doris Njomo, Charles Mwandawiro, Ulrike Fillinger, Rachel Pullan and Stella Kepha in Environmental Health Insights

sj-docx-5-ehi-10.1177_11786302241246454 – Supplemental material for Identifying Potential Determinants of Faecal Contamination on Domestic Floors in Three Settings in Rural Kenya: A Mixed Methods Analysis Supplemental material, sj-docx-5-ehi-10.1177_11786302241246454 for Identifying Potential Determinants of Faecal Contamination on Domestic Floors in Three Settings in Rural Kenya: A Mixed Methods Analysis by Hugo Legge, Karisa Kazungo, Sharon Muli, Lynne Elson, Jacinta Mwongeli, Katherine E Halliday, Victoria Ochwal, William Oswald, Robert Dreibelbis, Doris Njomo, Charles Mwandawiro, Ulrike Fillinger, Rachel Pullan and Stella Kepha in Environmental Health Insights

sj-docx-6-ehi-10.1177_11786302241246454 – Supplemental material for Identifying Potential Determinants of Faecal Contamination on Domestic Floors in Three Settings in Rural Kenya: A Mixed Methods Analysis Supplemental material, sj-docx-6-ehi-10.1177_11786302241246454 for Identifying Potential Determinants of Faecal Contamination on Domestic Floors in Three Settings in Rural Kenya: A Mixed Methods Analysis by Hugo Legge, Karisa Kazungo, Sharon Muli, Lynne Elson, Jacinta Mwongeli, Katherine E Halliday, Victoria Ochwal, William Oswald, Robert Dreibelbis, Doris Njomo, Charles Mwandawiro, Ulrike Fillinger, Rachel Pullan and Stella Kepha in Environmental Health Insights

sj-docx-7-ehi-10.1177_11786302241246454 – Supplemental material for Identifying Potential Determinants of Faecal Contamination on Domestic Floors in Three Settings in Rural Kenya: A Mixed Methods Analysis Supplemental material, sj-docx-7-ehi-10.1177_11786302241246454 for Identifying Potential Determinants of Faecal Contamination on Domestic Floors in Three Settings in Rural Kenya: A Mixed Methods Analysis by Hugo Legge, Karisa Kazungo, Sharon Muli, Lynne Elson, Jacinta Mwongeli, Katherine E Halliday, Victoria Ochwal, William Oswald, Robert Dreibelbis, Doris Njomo, Charles Mwandawiro, Ulrike Fillinger, Rachel Pullan and Stella Kepha in Environmental Health Insights

sj-docx-8-ehi-10.1177_11786302241246454 – Supplemental material for Identifying Potential Determinants of Faecal Contamination on Domestic Floors in Three Settings in Rural Kenya: A Mixed Methods Analysis Supplemental material, sj-docx-8-ehi-10.1177_11786302241246454 for Identifying Potential Determinants of Faecal Contamination on Domestic Floors in Three Settings in Rural Kenya: A Mixed Methods Analysis by Hugo Legge, Karisa Kazungo, Sharon Muli, Lynne Elson, Jacinta Mwongeli, Katherine E Halliday, Victoria Ochwal, William Oswald, Robert Dreibelbis, Doris Njomo, Charles Mwandawiro, Ulrike Fillinger, Rachel Pullan and Stella Kepha in Environmental Health Insights
