# Supplementary material for: Identifying Potential Determinants of Faecal Contamination on Domestic Floors in Three Settings in Rural Kenya: A Mixed Methods Analysis
Source: Environ Health Insights. 2024 May 10;18:11786302241246454. doi: 10.1177/11786302241246454 (PMC11088304; doi:10.1177/11786302241246454)
Supplement: sj-docx-1-ehi-10.1177_11786302241246454 – Supplemental material for Identifying Potential Determinants of Faecal Contamination on Domestic Floors in Three Settings in Rural Kenya: A Mixed Methods Analysis [file sj-docx-1-ehi-10.1177_11786302241246454.docx]

Field officer ____________________ Head of household _______________________________ village ____________________ date |__|__|/|__|__|/|__|__|

| 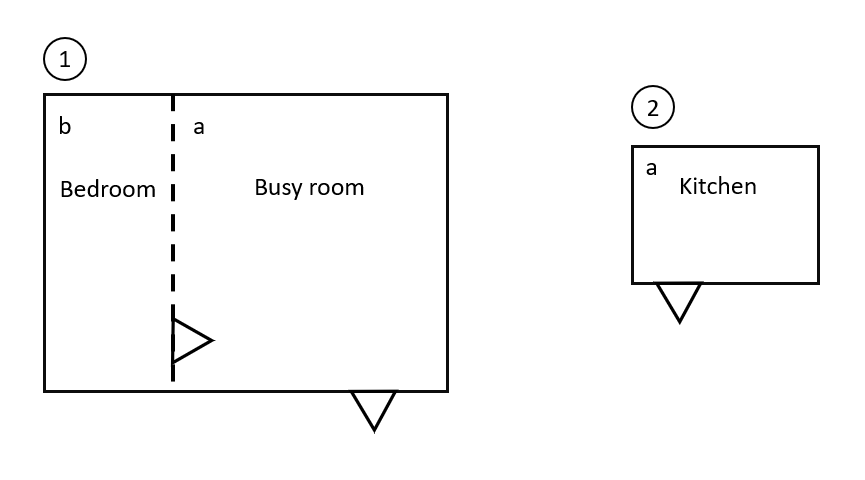 |
| --- |

|  |
| --- |

Field officer ____________________ Head of household _______________________________ village ____________________ date |__|__|/|__|__|/|__|__|

| **Building ID** | Main wall type of building | Main roof type of building | Age (years) | **Room ID** | Room name | Floor type | Reported uses for room (current) | Individuals who usually sleep in this room (list IDs) | Notes *(include description of “other” activities & seasonal variations in uses of room – if any)* |
| --- | --- | --- | --- | --- | --- | --- | --- | --- | --- |
| 1 | 1 2 3 4 5 6 | 1 2 3 4 5 | 5 | a | Busy room | 1 2 3 4 5 | 1 2 3 4 5 6 7 8 9 | 1 2 3 4 5 6 7 8 9 10 11 12 13 |  |
| 1 | 1 2 3 4 5 6 | 1 2 3 4 5 | 5 | b | Bedroom | 1 2 3 4 5 | 1 2 3 4 5 6 7 8 9 | 1 2 3 4 5 6 7 8 9 10 11 12 13 |  |
| 2 | 1 2 3 4 5 6 | 1 2 3 4 5 | 2 | A | Kitchen | 1 2 3 4 5 | 1 2 3 4 5 6 7 8 9 | 1 2 3 4 5 6 7 8 9 10 11 12 13 |  |
|  | 1 2 3 4 5 6 | 1 2 3 4 5 |  |  |  | 1 2 3 4 5 | 1 2 3 4 5 6 7 8 9 | 1 2 3 4 5 6 7 8 9 10 11 12 13 |  |
|  | 1 2 3 4 5 6 | 1 2 3 4 5 |  |  |  | 1 2 3 4 5 | 1 2 3 4 5 6 7 8 9 | 1 2 3 4 5 6 7 8 9 10 11 12 13 |  |
|  | 1 2 3 4 5 6 | 1 2 3 4 5 |  |  |  | 1 2 3 4 5 | 1 2 3 4 5 6 7 8 9 | 1 2 3 4 5 6 7 8 9 10 11 12 13 |  |
|  | 1 2 3 4 5 6 | 1 2 3 4 5 |  |  |  | 1 2 3 4 5 | 1 2 3 4 5 6 7 8 9 | 1 2 3 4 5 6 7 8 9 10 11 12 13 |  |
|  | 1 2 3 4 5 6 | 1 2 3 4 5 |  |  |  | 1 2 3 4 5 | 1 2 3 4 5 6 7 8 9 | 1 2 3 4 5 6 7 8 9 10 11 12 13 |  |
|  | 1 2 3 4 5 6 | 1 2 3 4 5 |  |  |  | 1 2 3 4 5 | 1 2 3 4 5 6 7 8 9 | 1 2 3 4 5 6 7 8 9 10 11 12 13 |  |

Wall type: 1-Sticks or pole with mud, 2-Stone with mud, 3-Cement, 4-Stone with cement, 5-No walls, 6-Other
Roof type: 1-Grass/palm thatch, 2-metal, 3-cement, 4-no roof, 5-Other
Floor type: 1-earth/clay/sand/soil, 2-wood/palm/bamboo, 3-Vinyl/plastic tiles, 4-Ceramic tiles, 5-concrete
Reported uses: 1-Sleeping, 2-Living/sitting, 3-Working, 4-Cooking/Preparing, 5-Dining/Eating, 6-Storage, 7-Keeping livestock, 8-wash room, 9-other
