# Supplementary material for: Identifying Potential Determinants of Faecal Contamination on Domestic Floors in Three Settings in Rural Kenya: A Mixed Methods Analysis
Source: Environ Health Insights. 2024 May 10;18:11786302241246454. doi: 10.1177/11786302241246454 (PMC11088304; doi:10.1177/11786302241246454)
Supplement: sj-docx-2-ehi-10.1177_11786302241246454 – Supplemental material for Identifying Potential Determinants of Faecal Contamination on Domestic Floors in Three Settings in Rural Kenya: A Mixed Methods Analysis [file sj-docx-2-ehi-10.1177_11786302241246454.docx]

On arrival introduce yourself to the household members, set up your camera and then with the help of the household members find a strategic place to sit where you can observe most of the activities happening in the household.

Throughout the course of the observation field officers should be taking detailed notes on specific activities of interest. These include:

- 1. **Floor hygiene practices –** *Any activity carried out on the floor of a household building or courtyard that is intended to remove observable or unobservable objects or contaminants. This includes picking up larger objects such as leaves or maize husks, as well as sweeping, mopping and washing.*
- Record the condition of the courtyard floor when you arrive at the dwelling
- Record the dwelling/space (Veranda or courtyard) that was cleaned
- Record the person who does the cleaning in each area
- Record the amount of time observed when cleaning is done in any observable dwelling
- Record the of type of object used in cleaning (reed/grass broom, twig broom, mop etc)

Describe any process followed during the cleaning e.g., springling water before sweeping, or mopping after sweeping. Record in numbered sequence

Describe what happens to dust and waste once it has been cleaned from the floor.

What happens to water that has been used to clean the floor

1. **Animal husbandry practices** – *Activities that involve direct or indirect interaction with animals (either livestock or pets) in the dwelling environment. This includes feeding animals, caging them, herding them, petting them etc. All animals present within the dwelling environment should be included in these observations, including livestock such as goats, chickens and cows, as well as dogs and cats.*

- List the observed type and number of animals within the household
- Record the people involved in animal husbandry activities
- State any observed interaction done to the animals e.g., feeding, watering, tethering herding
- Record the dwelling or space where the animals are kept or spends most of their time during your observation period
- Record if an animal defecates – where it is when it defecates
- Record if you can observe animal faeces on the courtyard floor during the observation period.
- Record if animals if you see animal enter any buildings e.g., chicken, dusk, goats

1. **Storing and preparing food and eating** – *Any activity that involves an interaction with food within the domestic environment – this includes the storing, cleaning, preparing (e.g. peeling and chopping), cooking and eating of food stuffs by household members. This does not include selling food to non-household members from a shop that is present within the dwelling.*

- List the dwelling/space where preparing food/cooking takes place
- Record the activities that accompany food preparation (chopping, peeling, grinding)
- where is unprepared and prepared food placed when food preparation is happening (e.g. in a bowl? On the ground?)
- Describe where people are when they are eating – what space are they in? are they sat on the ground or on a mat?
- Describe the eating process i.e., do people wash hands with soap before eating.
- Are utensils washed and used while still wet or had they been washed and dried prior
- Do people sit together while eating (communal eating), children fed first
- How are the used utensils stored/kept after eating?
- Describe the stove/fire that is used for cooking. What fuel is used?

1. **Child feces disposal and other caregiving activities** – *Activities that involve the provision of care to children (under the age of 5). Including feeding, washing, bathing, dressing, and emotional support activities. Particular attention should be paid to child defecation practices and processes for disposing of child feces and cleaning the child afterwards.*

- Number of children at dwelling during observation
- Record any bathing of children that takes place at the household and who carries this out
- Record the handling of the children faecal matter
- Who cleans the baby’s faecal matter?
- How is the baby cleaned (wiping or washing (plain water or with soap))
- Where are the faecal matter disposed? (garden/composite pit or toilet)
- How does the baby handler wash their hands after handling baby’s faecal matter?

1. **Hand/foot hygiene activities** – *Actions that are intended to remove observable and unobservable contaminants from hands and/or feet.*

- Do the household members have shoes (specify the type of shoes worn by different members of the household e.g., slippers, open flops, closed shoes)
- Observe handwashing sanitary facilities and practices (Water only, running water/water in a basin, running water with soap)
- Observe if people washed hands or feet and the events preceding the washing (Toilet visit, before eating).
- Observe if any household member encourages other household members to wash their hands
- If no handwashing or footwashing was observed through session – make record of this

1. **Water collection and storage** – *The collection, transportation, and storage of water from sources outside or within the dwelling to a place within the dwelling.*

- How was water acquired in the household? (Fetched by children or household member, running tap)
- Where is the water stored?
- Do the containers have covers?
