# Supplementary material for: Identifying Potential Determinants of Faecal Contamination on Domestic Floors in Three Settings in Rural Kenya: A Mixed Methods Analysis
Source: Environ Health Insights. 2024 May 10;18:11786302241246454. doi: 10.1177/11786302241246454 (PMC11088304; doi:10.1177/11786302241246454)
Supplement: sj-docx-3-ehi-10.1177_11786302241246454 – Supplemental material for Identifying Potential Determinants of Faecal Contamination on Domestic Floors in Three Settings in Rural Kenya: A Mixed Methods Analysis [file sj-docx-3-ehi-10.1177_11786302241246454.docx]

SABABU household observation write-up guide

1. Describe the condition of the floors and any floor hygiene activities that were observed over the two days – include:
   1. The cleanliness of the ground in the courtyard and any inside floors you were able to observe when you arrived at the dwelling and throughout the day
   2. What time you observed floor hygiene activities taking place, and in what spaces.
   3. Who was carrying out the activities
   4. What was the cleaning process
   5. How long it took
   6. Where any dust/dirt/food waste was disposed
   7. Any reflections/impressions on floor hygiene
2. Describe the animal husbandry activities that you observed over the two days – include:
   1. If you saw any animals being released from inside a building, shed or pen when you arrived – where they were released from.
   2. What animals were present in the dwelling during your observation
   3. Where animals were located during your observation period – if they free to roam or tied up – and if they were observed entering buildings.
   4. What interactions you observed taking place between household members and animals
   5. Which household members interacted with which animals
   6. Any reflections/impressions on animal husbandry
3. Describe any activities relating to the storing, preparing and eating of food that you observed over the two days – include:
   1. Where food preparation took place
   2. Which household members were involved in food preparation
   3. How food was stored while it was being prepared (e.g. placed in a bowl on the ground)
   4. What was the process for preparing and cooking food
   5. What type of fire was used (if observed) and what type of fuel.
   6. Where eating took place
   7. If handwashing was observed before or after preparing food or eating
   8. Where dishwashing took place
   9. Who performed the dishwashing
   10. What was done with the water used for dishwashing
   11. Any reflections/impressions on cooking/eating activities
4. Describe child caregiving practices for children less than five that you observed over the two days – include:
   1. Children under five that were present at the household during your visit
   2. Where children spent the majority of their time during your visit
   3. Any cleaning, washing, or bathing of children under five undertaken by other household members
   4. Where caregivers and children were when these activities occurred
   5. If feces was disposed of, where it was disposed, who disposed of it and if handwashing was undertaken before or afterwards
   6. Any impressions/reflections on child caregiving
5. Describe hygiene practices observed over the two days – Include:
   1. If handwashing was observed
   2. Which household members were observed washing their hands
   3. Where household members washed their hands
   4. What handwashing facilities were available
   5. If the washing of feet was observed
   6. Which household members were observed washing feet
   7. If household members were wearing shoes at any time
   8. Whether any pattern in shoe wearing was observed (e.g. household members wearing shoes in the courtyard but taking them off before entering a building)
   9. Any reflections/impressions on hygiene practices
6. Describe water collection and storage practices observed over the two days – Include:
   1. If you observed any household member bring water to the household
   2. Where was the water stored (if observed)
   3. In what containers was the water stored (if observed)
   4. If animals were observed as coming into contact with stored water (if observed)
   5. If you observed different water sources being used for different purposes
   6. If you observed household members treating water in anyway
   7. If you observed any rainwater harvesting infrastructure – and what condition this infrastructure was in
   8. Any reflections/impressions on water collection and storage
7. Describe any laundry practices that were observed over the two days – Include:
   1. If washing of clothes was observed
   2. Where washing of clothes was observed as taking place
   3. Who was observed washing clothes
   4. What water was used and what happened to the water afterwards
   5. Where were clothes hung to dry?
   6. Any reflections/impressions on laundry practices
8. If the household is located in a boma, summarise how households in the same boma interacted – include:
   1. How much time did members from this household spend in other dwellings in the same boma
   2. If any activities were undertaken together (for example, dishes from all households are washed together) what were these activities and where were they undertaken.
   3. If tools, materials, or food were observed as being shared between households.
   4. Any reflections/impressions households living together in a boma
9. Summarise how observed space/s were used and how busy they were over the two days – Include:
   1. Which household members were present at the household and for how long during AM and PM observations on day 1 and day 2
   2. Which building/space household members were observed spending time in and impressions on how busy these buildings/spaces were during AM and PM observations on day 1 and day 2
   3. Impression on which building/space was the “busy” space (the space where most of the activities were carried out).
   4. Any other impressions on how buildings/spaces were used during observations – including any observed difference between reported uses for a building and observed uses
10. Describe what the household’s reaction was to your presence and the presence of the camera on both days of observations – Include:
    1. How you feel you were received by household members. What was the reaction to your presence and the presence of the camera?
    2. Did you observe a difference between how you were received on the first and second day of observations?
    3. Any interaction that you had with household members that you think is note worthy
