# Supplementary material for: Identifying Potential Determinants of Faecal Contamination on Domestic Floors in Three Settings in Rural Kenya: A Mixed Methods Analysis
Source: Environ Health Insights. 2024 May 10;18:11786302241246454. doi: 10.1177/11786302241246454 (PMC11088304; doi:10.1177/11786302241246454)
Supplement: sj-docx-4-ehi-10.1177_11786302241246454 – Supplemental material for Identifying Potential Determinants of Faecal Contamination on Domestic Floors in Three Settings in Rural Kenya: A Mixed Methods Analysis [file sj-docx-4-ehi-10.1177_11786302241246454.docx]

**SABABU Household observation video summary form**

| Reviewer name |  | | |
| --- | --- | --- | --- |
| Household ID |  | Morning | |
| County |  | Time start (AM) |  |
| Date of observation |  | Time end (AM) |  |
| Day # at household |  | Duration (AM) |  |
| Room name |  | Afternoon | |
| Room ID |  | Time start (PM) |  |
| Number of clips (AM/PM) |  | Time end (PM) |  |
| Camera ID |  | Duration (PM) |  |

| Camera setting and visible scene |
| --- |
| *Describe the view and location of the camera during AM and PM* |
| AM:  PM: |

| Floor condition and hygiene activities |
| --- |
| *Describe the condition of the floors and any floor hygiene activities that were observed* |
|  |

| Animal husbandry and animal movement | |
| --- | --- |
| Number of clips animals present in: |  |
| *Describe the animal husbandry activities that were observed and animal movement* | |
|  | |

| Food |
| --- |
| *Describe any activities relating to the storing, preparing, eating of food and washing utensils* |
|  |

| Child caregiving |
| --- |
| *Describe child caregiving practices for children less than five that you observed – and actions of children under five* |
|  |

| Hygiene |
| --- |
| *Describe hygiene practices observed, including foot and hand hygiene. Also shoe wearing practices* |
|  |

| Water |
| --- |
| *Describe water collection and storage practices observed* |
|  |

| Laundry |
| --- |
| *Describe any laundry practices that were observed* |
|  |

| Reactivity |
| --- |
| *Describe any impressions of reactivity that you observed* |
|  |

| Conclusions/reflections |
| --- |
| *Describe overall impression of how the spaced was used, how busy it was, who used the space and what their responsibilities appeared to be* |
|  |
