# Supplementary material for: Identifying Potential Determinants of Faecal Contamination on Domestic Floors in Three Settings in Rural Kenya: A Mixed Methods Analysis
Source: Environ Health Insights. 2024 May 10;18:11786302241246454. doi: 10.1177/11786302241246454 (PMC11088304; doi:10.1177/11786302241246454)
Supplement: sj-docx-5-ehi-10.1177_11786302241246454 – Supplemental material for Identifying Potential Determinants of Faecal Contamination on Domestic Floors in Three Settings in Rural Kenya: A Mixed Methods Analysis [file sj-docx-5-ehi-10.1177_11786302241246454.docx]

Floor Hygiene and sleeping arrangements

1. Can you describe what you do to keep the courtyard ground clean within your home?
   1. **Prompt –** Who’s responsibility is it to do this?
   2. **Prompt –** How long does this normally take?
   3. **Prompt –** What prompts you to clean this ground?
   4. **Prompt –** What do you do with the dust/dirt?
2. Which rooms are used for sleeping (mention floor and the name of the dwelling)?
   1. What do people sleep on? (Probe for whether there is a bed)
3. Are there variations in sleeping arrangements according to the time of year *(e.g. school period, harvest period, rainy season, dry season)*?
4. Now thinking about building (x), can you describe the routines that you are currently following to keep the floors clean within your home?
5. **Prompt –** Who’s responsibility is it to do this?
6. **Prompt –** How long does this normally take?
7. **Prompt –** What prompts you to clean this floor?
8. **Prompt –** What do you do with the dust/dirt?
9. **Prompt –** Do you use water to clean the floor? What do you do with the water afterwards?
10. Thinking about different times in the year, do your cleaning routines change according to the season *(e.g. school period, harvest period, rainy season, dry season)?*
11. What can make a floor dirty?

Animal husbandry

1. Thinking about the different animals that you own, what do you do to take care of them?
   1. **Prompt** – What are the feeding practices?
   2. **Prompt** – How do you water them?
2. Do animals go inside during the day (including cats and/or dogs)? If no, why not? If yes, why?
3. Where do you keep your animals at night?
   1. If inside, why do you choose to keep your animals inside during the night?
   2. How often do you clean their sleeping areas?
4. Do you think its better for chickens to roam freely, or to be put in a chicken house?
   1. <If better to roam freely> why do you think this?
   2. <If better in a chicken house> why do you think this?
5. Do animal husbandry routines change according to the time of year *(e.g. school period, harvest period, rainy season, dry season)*? If yes, describe the changes.

Child caregiving

1. How many children under five do you have? How old are they?
2. Where does each child (under five) defecate?
   1. What do you usually do to clean them? Do you use anything?
   2. Where do you dispose of the child’s faeces?

Can you please tell me what hygiene practices you take after cleaning your baby’s fecal matter?

Water collection and use

1. Can you describe how water is collected and brought to the house?
   1. **Prompt –** Where do you collect water from?
   2. **Prompt –** Who collects the water?
   3. **Prompt –** How long does it take?
   4. **Prompt –** Do you pay for water?
2. Do water collection routines vary according to the time of year *(e.g. school period, harvest period, rainy season, dry season)*? If so, how?
3. How do you store water when it’s at the home?
   1. **Prompt** – Do you have different water stores that you use for different tasks? If yes, why do you have different stores?
4. Do you do anything to your drinking water to make it safer to drink? If yes, why do you choose to treat your water like this?

Food preparation

1. Can you describe where you store your food? Why do you choose to store your food here?
2. **Prompt –** *is there a difference between storage for cooked and uncooked food*
3. Where do you carry out food preparation?
   1. (If they prepare food outside) Why do you prepare food outside?
4. Is your cooking fire/stove inside or outside?
   1. Why do you choose to have it in this place?
5. Do food storage or cooking routines change according to the time of year *(e.g. school period, harvest period, rainy season, dry season)*? If yes, how and why?

Health

1. What are the main three diseases affecting people in this community/village?
2. (If jiggers not mentioned by respondent) Are jiggers a problem for your family?
3. (If jiggers not a problem for family) Do you think jiggers are a problem in this community?

*Only proceed with following question if jiggers are cited as being either a problem for the family or a problem in the community more generally:*

1. What are the causes of jigger infections in the community?
   1. Are there places around your home, or the village that you avoid because you know jiggers are there?
2. Are jiggers worse at different points in the year? If yes, which seasons are bad for jiggers*?*
3. How can jigger infections be treated or prevented*?*
