# Supplementary material for: Identifying Potential Determinants of Faecal Contamination on Domestic Floors in Three Settings in Rural Kenya: A Mixed Methods Analysis
Source: Environ Health Insights. 2024 May 10;18:11786302241246454. doi: 10.1177/11786302241246454 (PMC11088304; doi:10.1177/11786302241246454)
Supplement: sj-docx-6-ehi-10.1177_11786302241246454 – Supplemental material for Identifying Potential Determinants of Faecal Contamination on Domestic Floors in Three Settings in Rural Kenya: A Mixed Methods Analysis [file sj-docx-6-ehi-10.1177_11786302241246454.docx]

Household case memo

| HH ID |  | Group |  |
| --- | --- | --- | --- |
| Site |  | Reviewer |  |
| Village |  | Review completed |  |

| Member number | Sex | Age | Head of household |
| --- | --- | --- | --- |
| 1 |  |  |  |
| 2 |  |  |  |
| 3 |  |  |  |
| 4 |  |  |  |
| 5 |  |  |  |
| 6 |  |  |  |

| Dwelling layout and household summary |
| --- |
|  |

| Floor hygiene |
| --- |
| Summary of courtyard/veranda  Summary of inside buildings  Selected excerpts from transcripts  Impressions |

| Food preparation and eating |
| --- |
| Food Preparation  Cooking  Cleaning utensils  Eating  Selected excerpts from transcripts  Impressions |

| Animal husbandry |
| --- |
| Overview of animals in household  Sleeping arrangements + cleaning of sleeping area  Animal handling / Interaction of animals without household members  Selected excerpts from transcripts  Impressions |

| Sleeping arrangements |
| --- |
| What rooms for sleeping  Apparatus for sleeping  Seasonal variation  Selected excerpts from transcripts  Impressions |

| Child caregiving |
| --- |
| Approaches for child minding/handling  Where children spent time  Where they defecate + hygiene practices |

| Water storage and collection |
| --- |
| Different uses for water/different stores (include variation in season)  Sources  Time and cost  whose responsibility |

| Hygiene |
| --- |
| Handwashing  Footwashing  Shoe wearing |

| Laundry |
| --- |
| Who is responsible  Where is it conducted  How long does it take  Where water disposed |

| Health |
| --- |
| Major health concerns in community  Presence of jiggers in community and causes  Prevention of jiggers and any places avoided  Treatment of jiggers |
