# Supplementary material for: Identifying Potential Determinants of Faecal Contamination on Domestic Floors in Three Settings in Rural Kenya: A Mixed Methods Analysis
Source: Environ Health Insights. 2024 May 10;18:11786302241246454. doi: 10.1177/11786302241246454 (PMC11088304; doi:10.1177/11786302241246454)
Supplement: sj-docx-7-ehi-10.1177_11786302241246454 – Supplemental material for Identifying Potential Determinants of Faecal Contamination on Domestic Floors in Three Settings in Rural Kenya: A Mixed Methods Analysis [file sj-docx-7-ehi-10.1177_11786302241246454.docx]

Table S1. Household characteristics in the Bungoma, Kwale, and Narok study settings

|  |  | **Kwale**  **(N=812)** | **Bungoma (N=906)** | **Narok**  **(N=1102)** |
| --- | --- | --- | --- | --- |
| **Variable** | **Category** | **n (%)** | **n (%)** | **n (%)** |
| Household size | Mean (SD) | 6.4 (3.2) | 5 (2.3) | 4.5 (2) |
| Education of Head of household | No education | 234 (28.8) | 78 (8.6) | 847 (76.9) |
|  | Primary education | 414 (51) | 505 (55.8) | 172 (15.6) |
|  | Secondary education or above | 164 (20.2) | 322 (35.6) | 83 (7.5) |
| Number of buildings in household | 1 building | 219 (27) | 272 (30.1) | 815 (74) |
|  | 2 buildings | 282 (34.7) | 415 (45.9) | 259 (23.5) |
|  | 3 buildings | 179 (22) | 162 (17.9) | 21 (1.9) |
|  | 4+ buildings | 132 (16.3) | 56 (6.2) | 7 (0.6) |
| Floor type | Earthen floor (all buildings) | 608 (74.9) | 679 (75) | 972 (88.2) |
|  | Improved floor in part/all of household | 204 (25.1) | 226 (25) | 130 (11.8) |
| Primary water source type | Surface water | 19 (2.3) | 2 (0.2) | 941 (85.4) |
|  | Unimproved source† | 247 (30.4) | 128 (14.2) | 35 (3.2) |
|  | Improved source* | 539 (66.4) | 771 (85.3) | 118 (10.7) |
|  | Piped on to plot | 7 (0.9) | 3 (0.3) | 8 (0.7) |
| Return journey time to water source (m) | 1-5 minutes | 185 (23.4) | 167 (18.6) | 84 (7.7) |
|  | 5-30 minutes | 468 (59.3) | 606 (67.4) | 638 (58.3) |
|  | 30-60 minutes | 120 (15.2) | 122 (13.6) | 234 (21.4) |
|  | more than 1 hour | 16 (2) | 4 (0.4) | 138 (12.6) |
| Sanitation access | No facility/field | 89 (11) | 31 (3.4) | 887 (80.6) |
|  | Access to unimproved facility | 108 (13.4) | 148 (16.4) | 14 (1.3) |
|  | Access to improved facility | 611 (75.6) | 724 (80.2) | 200 (18.2) |
| Sanitation sharing status | Exclusive access to facility | 474 (58.4) | 519 (57.3) | 133 (62.3) |
|  | Sharing access with other households | 338 (41.6) | 386 (42.7) | 81 (37.7) |
| Ownership of any animal^#^ | No animal owned | 123 (15.2) | 178 (19.7) | 197 (17.9) |
|  | At least one animal owned | 689 (84.9) | 727 (80.3) | 905 (82.1) |
| Number of poultry owned^^^ | None | 224 (27.6) | 247 (27.3) | 694 (63) |
|  | 1-5 animals | 202 (24.9) | 344 (38) | 149 (13.5) |
|  | 6-20 animals | 314 (38.7) | 284 (31.4) | 246 (22.3) |
|  | 20+ animals | 72 (8.9) | 30 (3.3) | 13 (1.2) |
| Number of goats/sheep owned | None | 400 (49.3) | 718 (79.3) | 305 (27.7) |
|  | 1-5 animals | 274 (33.7) | 180 (19.9) | 74 (6.7) |
|  | 6-20 animals | 128 (15.8) | 7 (0.8) | 236 (21.4) |
|  | 20+ animals | 10 (1.2) | 0 (0) | 487 (44.2) |
| Number of cattle owned | None | 468 (57.6) | 468 (51.7) | 327 (29.7) |
|  | 1-5 animals | 223 (27.5) | 408 (45.1) | 230 (20.9) |
|  | 6-20 animals | 114 (14) | 28 (3.1) | 313 (28.4) |
|  | 20+ animals | 7 (0.9) | 1 (0.1) | 232 (21.1) |

*****Including borehole, public tapstand, and protected dug well
†Including unprotected dug well, unprotected spring, and rainwater

^#^Includes cattle, goats, sheep, any type of poultry, horses, donkeys, mules, pigs, and rabbits.

^^^Less than 2.5% of households owned horses, donkeys, mules, pigs, or rabbits so their ownership numbers are not provided in detail
