# Supplementary material for: Identifying Potential Determinants of Faecal Contamination on Domestic Floors in Three Settings in Rural Kenya: A Mixed Methods Analysis
Source: Environ Health Insights. 2024 May 10;18:11786302241246454. doi: 10.1177/11786302241246454 (PMC11088304; doi:10.1177/11786302241246454)
Supplement: sj-docx-8-ehi-10.1177_11786302241246454 – Supplemental material for Identifying Potential Determinants of Faecal Contamination on Domestic Floors in Three Settings in Rural Kenya: A Mixed Methods Analysis [file sj-docx-8-ehi-10.1177_11786302241246454.docx]

Routes of faecal contamination on domestic floors in three settings in rural Kenya: a mixed methods analysis

**Supplementary materials contents**

Document S1 – Observation floorplan and room index

Document S2 – Observational checklist guide

Document S3 – Observation write-up guide

Document S4 – Video summary guide

Document S5 – In-depth interview guide

Document S6 – Household case memo template

Document S7 – Household characteristics in the Bungoma, Kwale, and Narok study settings
